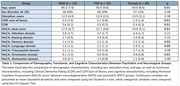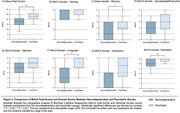# Cognitive Outcomes in Psychiatric Ward: Preliminary Results

**DOI:** 10.1002/alz70857_102001

**Published:** 2025-12-24

**Authors:** Alessandra Santini, Barbara Suman Bahlis, Fernando Jacob Lazzaretti, Lucas Porcello Schilling, Cristiano Aguzzoli

**Affiliations:** ^1^ Psychiatry Department, São Lucas Hospital of PUCRS, Porto Alegre, Rio Grande do Sul, Brazil; ^2^ School of Medicine, Pontifícia Universidade Católica do Rio Grande do Sul (PUCRS), Porto Alegre, Rio Grande do Sul, Brazil; ^3^ Brain Institute of Rio Grande do Sul, PUCRS, Porto Alegre, RS, Brazil; ^4^ Global Brain Health Institute, San Francisco, CA, USA; ^5^ Pontifícia Universidade Católica do Rio Grande do Sul, Porto Alegre, Brazil; ^6^ Brain Institute of Rio Grande do Sul (InsCer), Porto Alegre, Rio Grande do Sul, Brazil; ^7^ Neurology Department, São Lucas Hospital of PUCRS, Porto Alegre, Rio Grande do Sul, Brazil; ^8^ Global Brain Health Institute (GBHI), San Francisco, CA, USA

## Abstract

**Background:**

Studies conducted in high‐income countries show that a significant proportion of people admitted to psychiatric wards have an underlying neurodegenerative disease (NDD) associated with psychiatric (PSY) disorders. In fact, neuropsychiatric symptoms are associated with neuroinflammation in the context of an NDD can often mimic psychiatric disorders, leading to misdiagnosis. This study aims to investigate features and patterns in cognitive performance associated with PSY and NDD conditions in individuals admitted to a psychiatric unit real‐world clinical setting.

**Method:**

This is a prospective observational cohort study of patients admitted to a psychiatric ward. We assess functional status and determine whether individuals meet clinical diagnostic criteria for NDD during hospitalization. The target population includes patients over 45 years of age. Statistical analysis were performed by welch t‐test and chi‐squared test to estimate demographic differences between groups, and Analysis of Variance (ANOVA) for investigating cognitive domains within MoCA (Montreal Cognitive Assessment) test.

**Result:**

PSY group exhibits a higher proportion of women, a younger mean age, and a higher level of education compared to the NDD. Regarding cognitive performance, the PSY group demonstrates a higher mean MoCA total score (mean difference 4.8, 95% CI 1.45 to 8.06, *p* < 0.01). PSY group shows a lower mean score in the CDR assessment compared to the NDD group (mean difference ‐0.3, 95% CI ‐0.49 to ‐0.15, *p* < 0.01) (Table 1).

Our results further demonstrate a significant difference in memory (mean difference 1.1, 95% CI 0.14 to 2.0, *p* < 0.05), language (mean difference 0,7, 95% CI 0.01 to 1.52, *p* < 0.05), and orientation (mean difference 0.8, 95% CI 0.30 to 1.27, *p* < 0.01) within MoCA domains between the groups (Figure 2).

**Conclusion:**

As expected, our study revealed that NDD group presents worse cognitive and functional performance compared to individuals with PSY illness. Regarding cognitive domains, we found a significant difference in memory, language, and orientation domains in the degeneration process. These results highlight the importance of specific assessments to characterize individuals admitted to psychiatric ward that will be better detailed in further analysis.